# Comparative study on differentiation of cervical-loop cells and Hertwig’s epithelial root sheath cells under the induction of dental follicle cells in rat

**DOI:** 10.1038/s41598-018-24973-0

**Published:** 2018-04-25

**Authors:** Yongwen Guo, Weihua Guo, Jie Chen, Ye Tian, Guoqing Chen, Weidong Tian, Ding Bai

**Affiliations:** 1 State Key Laboratory of Oral Diseases, West China Hospital of Stomatology, Sichuan University, Chengdu, 610041 P.R. China; 2 National Engineering Laboratory for Oral Regenerative Medicine, West China Hospital of Stomatology, Sichuan University, Chengdu, 610041 P.R. China; 3 Department of Orthodontics, West China Hospital of Stomatology, Sichuan University, Chengdu, 610041 P.R. China; 4 Department of Pediatric Dentistry, West China Hospital of Stomatology, Sichuan University, Chengdu, P.R. China; 5 Department of Oral and Maxillofacial Surgery, West China Hospital of Stomatology, Sichuan University, Chengdu, 610041 P.R. China

## Abstract

Cervical loop cells (CLC) and Hertwig’s epithelial root sheath (HERS) cells are believed to play critical roles in distinct developmental patterns between rodent incisors and molars, respectively. However, the differences in differentiation between CLC and HERS cells, and their response to inductions from dental follicle cells, remain largely unknown. In present study, CLC and HERS cells, as well as incisor dental follicle (IF) cells and molar dental follicle (MF) cells were isolated from post-natal 7-day rats. IF and MF cell derived conditioned medium (CM) was obtained for induction of CLC and HERS cells. *In vitro* experiments, we found that, under the induction of dental follicle cell derived CM, CLC cells maintained the epithelial polygonal-shapes and formed massive minerals, while part of HERS cells underwent shape transformation and generated granular minerals. CLC cells expressed higher enamel-forming and mineralization related genes, while HERS cells showed opposite expression patterns of BMP2, BMP4, AMBN and AMGN. *In vivo*, CLC cells generated enamel-like tissues while HERS cells formed cementum-periodontal ligament-like structures. Taken together, CLC and HERS cells present distinct differentiation patterns under the inductions from dental follicle cells.

## Introduction

Rodent incisors and molars present two totally different developmental patterns. The incisors grow and erupt continuously the whole life without the formation of typical root, while the molars form typical roots after the completion of crown and stop erupting spontaneously after the completion of root development^1^. Tummers *et al*. believes that crown would be continuously generated and erupt if the cervical loop structure formed at the bell stage of the tooth germ was maintained. On the contrary, if the cervical loop structure was disrupted by induction of the modulating signals, forming a bilayer epithelial structure, known as Hertwig’s epithelial root sheath (HERS), initiation of the root development would occur^2^. Morphologically, both the cervical loop and HERS are structural boundaries of two dental mesenchymal tissues: dental papilla and dental follicle. The interactions between epithelial cells in the cervical loop or HERS structures and their adjacent mesenchymal cells play crucial roles in the differentiation and maturation of dental cells^3^.

The cervical loop is constantly maintained at the labial aspect of the rodent incisor apex and is the reservoir of stem cells. It consists of a core of stellate reticulum cells surrounded by inner and outer enamel epithelial cells that contact the dental mesenchyme. Harada *et al*.^4^ have reported that there are stem cells in the cervical loop and they are able to give rise to enamel forming ameloblasts. Indeed, the cells in the apex of the rodent incisors divide rapidly compared with the more incisal region, and there is a gradient of cell differentiation from the apex towards the incisal direction. The dental mesenchyme surrounding the cervical loop provides the vital molecular signals for maintaining of the cervical loop at the labial apex of the rodent incisors. As a matter of fact, Yang *et al*. have found that mesenchymal TGF-β signaling provides a unifying mechanism for the homeostasis of dental epithelial stem cells via a Wnt signaling-mediated mesenchymal-epithelial cell interaction^5^. Thesleff *et al*. also have concluded that the mesenchyme surrounding the cervical loop induces Fgf10 expression, which is negatively regulated by Wnt/β-catenin, to limit the apoptosis of mouse incisor epithelial cells^6^. Therefore, the cervical loop, a reservoir for ameloblasts, is maintained throughout life by interaction with surrounding mesenchyme. However, the impact of the surrounding dental follicle cells on cervical loop cells still needs further study.

HERS formation requires the disruption of cervical loop structure and marks the ignition of tooth root formation. In human teeth and rodent molars, after completion of crown, the inner and outer epithelium of enamel organ proliferate at the cervical loop and the stratum intermedium and stellate reticulum disappear to form HERS. It migrates apically and participates in root formation. Regarding the mechanisms of HERS in the formation of periodontium, some researchers conclude that HERS indirectly involves in the development of periodontal structures by interacting with the surrounding dental follicles, which contain precursors that give rise to the components of the periodontium including cementum, periodontal ligament and alveolar bone^7–9^. Some other researchers suggest that HERS cells undergo epithelial-mesenchymal transformation (EMT) to differentiate into cementoblasts and periodontal ligament fibroblasts participating in the formation of acellular cementum and part of periodontal ligament fibers^7,10^. There are also some studies claim that HERS cells involve in neither EMT nor direct formation of cementum and periodontal ligament fibers^9^. Therefore, it needs further investigation to clarify whether HERS directly or indirectly participate in the formation of cenmentum and periodontal ligament.

Many studies have reached the agreement that cervical loop and HERS are accountable for the differential development modes between rodent incisors and molars^2^. It is generally thought that cervical loop and HERS function as the signaling centres to direct the enamel formation or the root development in rodent incisors and molars, respectively^11,12^. However, the role and the difference between the two epithelial cells and their interaction with the dental mesenchyme remain largely unknown. Thus, the present study aimed to explore the differences in differentiation between CLC and HERS cells under the inductions from the dental follicle cells. The implementation of the investigation would further contribute to elucidate the mechanism of tooth development and provide experimental evidence for potential clinical application in tooth regeneration.

## Results

### Cell culture and identification

**Figure 1:**
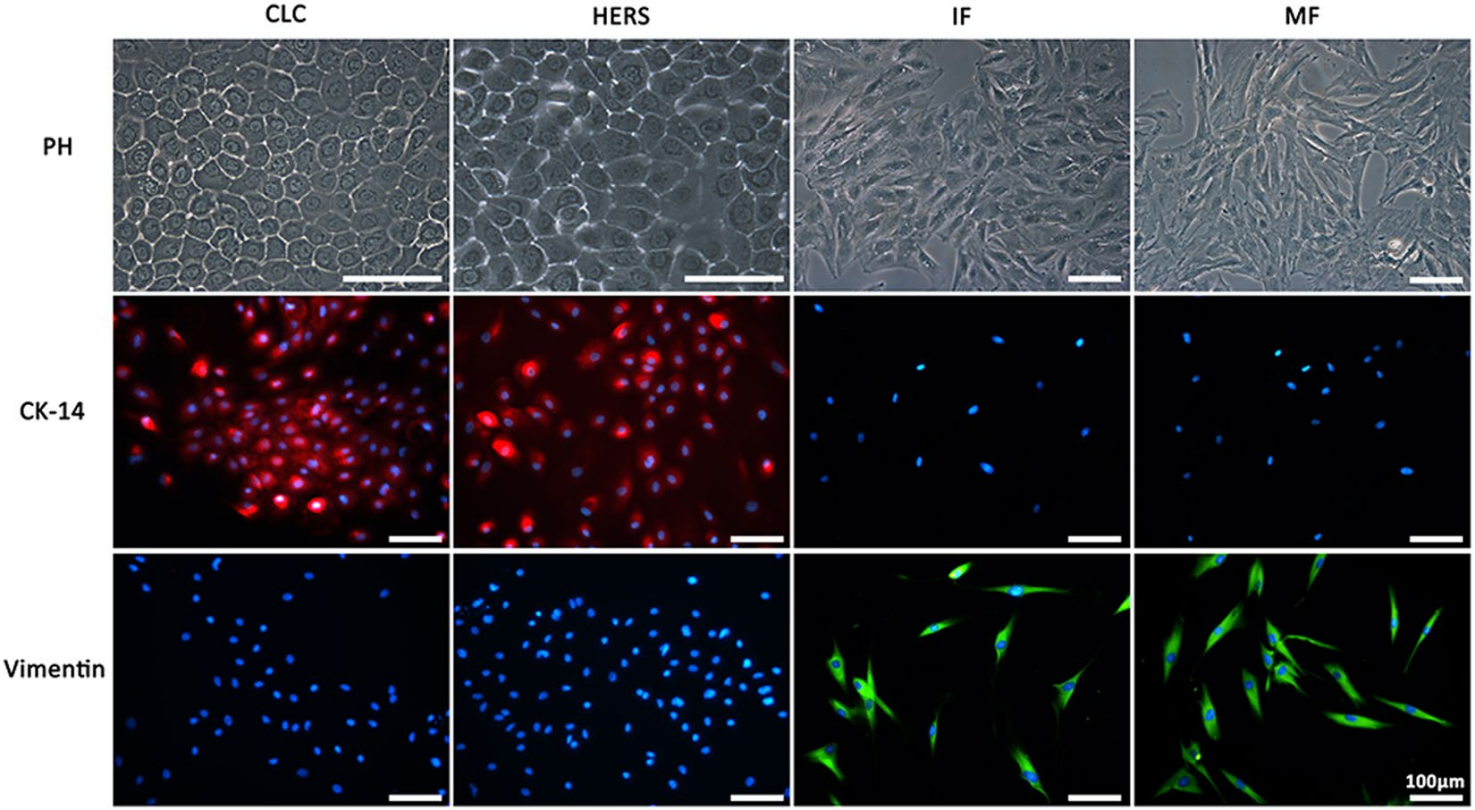
Purified CLC, HERS, IF and MF cells observed under phasecontrast microscope (PH) and identified by immunoinfluence staining. CLC and HERS cells were positive for CK-14 but negative for vimentin; IF and MF cells showed the opposite staining of CK-14 and vimentin to CLC and HERS cells.

Purified CLC and HERS cells as well as IF and MF cells of rats were obtained after 2 times of differential trypsin digestion. Both CLC and HERS cells connected tightly forming a typical polygonal-shape and paving stone-like appearance with multilayered growth after 3–5 days of culture. CLC cells were more cuboidal while the HERS cells were more ovoidal. IF and MF cells were characterized by a typical fibroblast-like morphology of spindle or stellate shape. Both CLC and HERS cells were positive for the epithelial cell marker CK14, but negative for the mesenchymal cell marker vimentin, while the IF and MF cells showed the opposite (Fig. 1). These results indicated that the methods to isolate CLC, HERS, IF and MF cells are effective with a high purification.

### CLC and HERS cells exhibited different morphologies and proliferation ability

**Figure 2:**
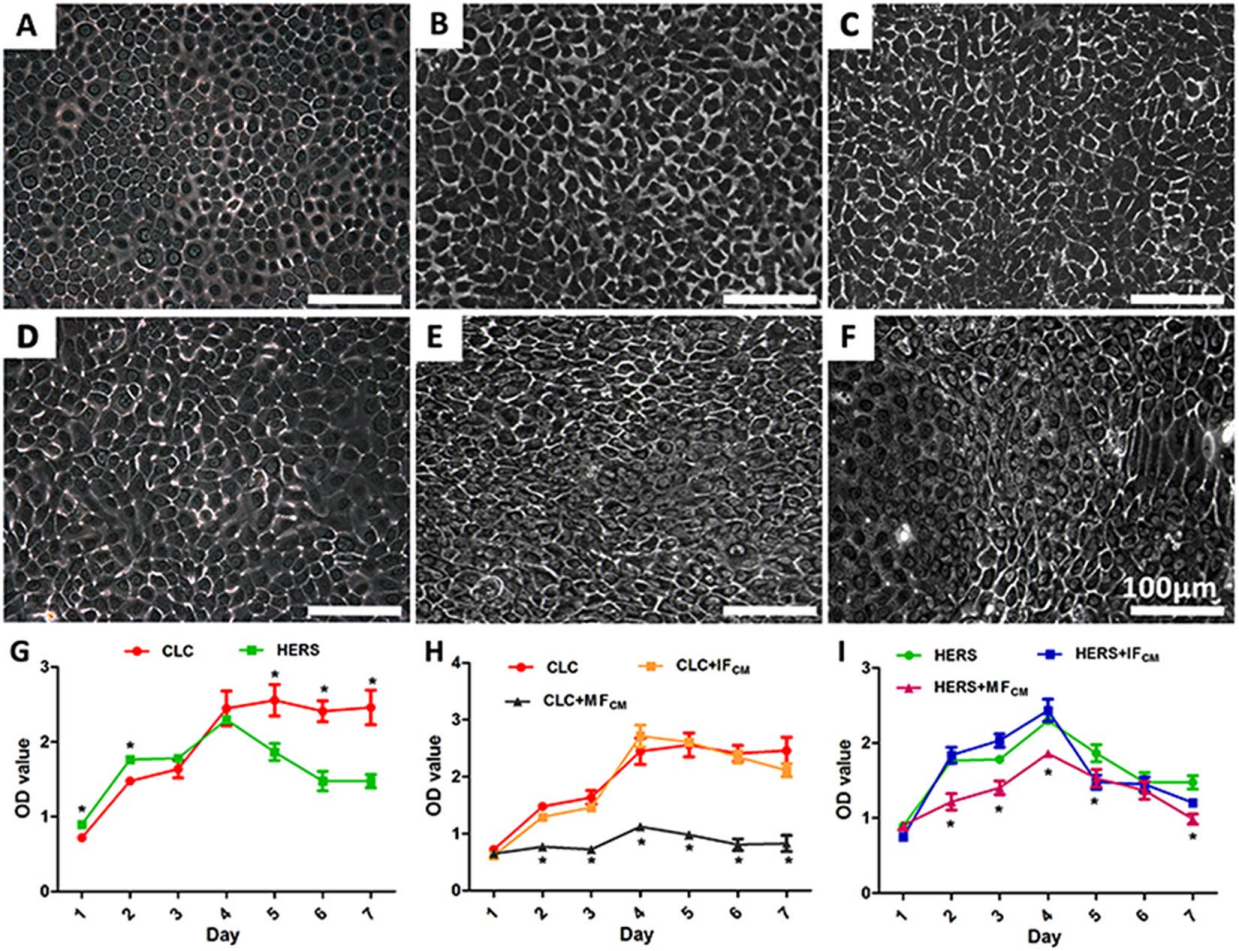
Morphological observation and CCK-8 analysis of CLC and HERS cells after induction by IF_CM_ and MF_CM_. Compared with non-induced CLC (**A**) and HERS cells (**D**), CLC induced with IF_CM_ (**B**) and MF_CM_ (**C**) maintained the polygonal-shape of epithelial cells, while part of HERS cells lost the epithelial cell shape and transformed into spindle-shaped cells (E,F). Morphologically transformed cells were more prominent in HERS cells induced by MF_CM_ (**F**) than IF_CM_ (**E**). CCK-8 analysis showed that non-induced CLC and HERS cells presented similar proliferation rate in the beginning and reached the peak at the 4^th^ day; thereafter, the number of CLCs maintained at the peak level while the number of HERS cells started decreasing (**G**). The proliferation of CLC cells were significantly inhibited by the MF_CM_ but not affected by the IF_CM_ (**H**), and so did the HERS cells with a moderate effect (**I**). Scale bars: 100 μm; *statistical difference found between groups with a significance level of p ≤ 0.05.

CLC cells maintained the polygonal-shapes of epithelial cells with or without the inductions by dental follicle cell derived CM, while part of HERS cells lost the epithelial cell shape and transformed into spindle-shaped cells after induction by the CM. The transformed HERS cells showed elongated cell body and nucleus, which resembled to the mesenchymal cells, and they were more prominent in HERS cells induced by MF_CM_ than IF_CM_ (Fig. 2A–F). CCK-8 analysis showed that when cultured with only EpiCM, both CLC and HERS cells proliferated in a similar rate and reached the peak at the 4^th^ day; the number of CLCs maintained at the peak level thereafter while the number of HERS cells started decreasing (Fig. 2G). The proliferation of CLC cells was significantly inhibited by the MF_CM_ induction but not affected by the IF_CM_ induction (Fig. 2H), and so did the HERS cells with a moderate effect (Fig. 2I).

### CLC and HERS cells produced different forms and amount of the minerals

**Figure 3:**
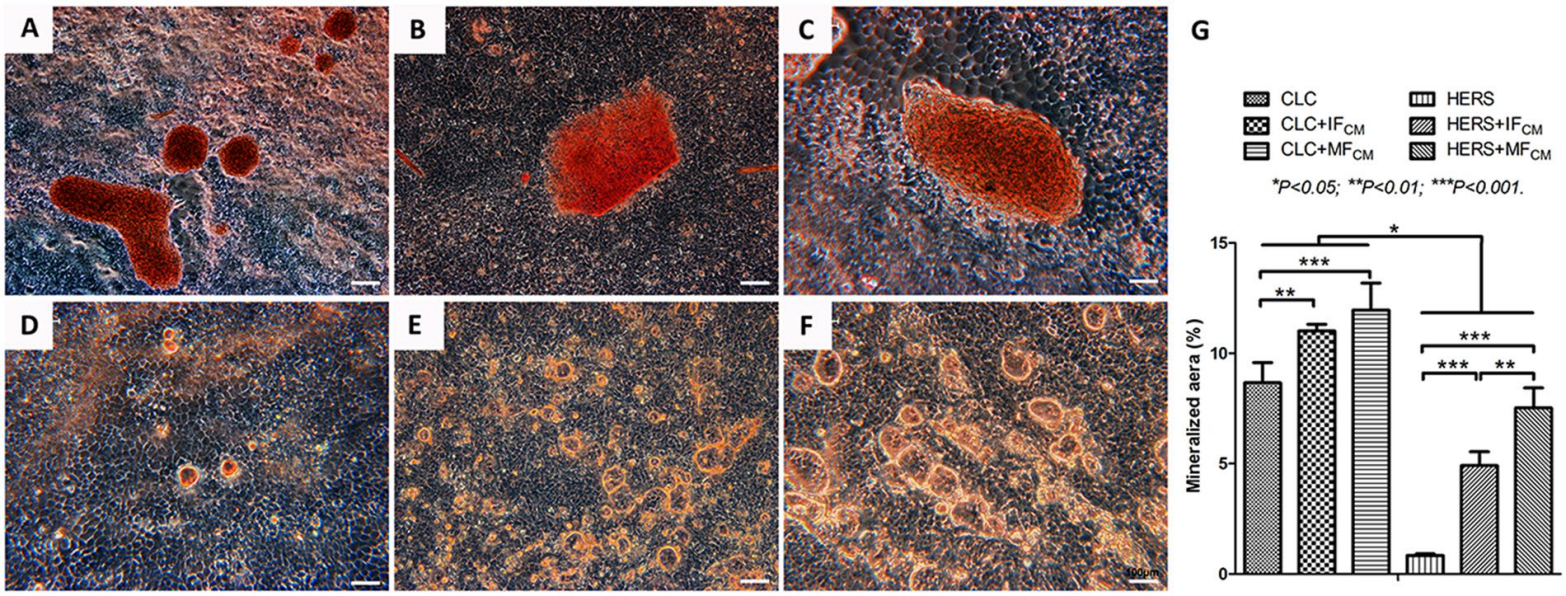
Alizarin red staining of CLC and HERS cells cultured with or without conditioned medium for 1 week. Massive minerals were found in CLC groups cultured with only EpiCM (**A**), or IF_CM_ (**B**) or MF_CM_ (**C**) while granular minerals were found in HERS cell groups (**D**: EpiCM; **E**: IF_CM_; **F**: MF_CM_). CLC cells owned the ability of mineral formation (A) while HERS cells hardly mineralized (D) when cultured without induction. After induction with IF_CM_ and MF_CM_, both CLC and HERS cells presented significant higher mineral formation than non-induced CLC and HERS cells. MF_CM_ induced the most abundant mineralization in both CLC and HERS cells. Beside, mineralizations in all three CLC cell groups were significantly higher than those in HERS cell groups (**G**). Scale bars: 100 μm.

Alizarin red staining showed different forms and amount of the minerals between CLC cells and HERS cells (Fig. 3). Massive minerals were formed in CLC groups while granular minerals were found in HERS cell groups. CLC cells owned the ability of mineral formation while HERS cells hardly produce minerals when cultured without induction. After induction with IF_CM_ and MF_CM_, both CLC and HERS cells presented significant higher mineral formation than non-induced CLC and HERS cells, and MF_CM_ induced the most abundant mineralization in both CLC and HERS cells. Besides, mineralizations in all CLC cell groups were significantly higher than those in HERS cell groups (Fig. 3G).

### CLC and HERS cells showed differential expression patterns of mineralization and enamel-forming related genes

**Figure 4:**
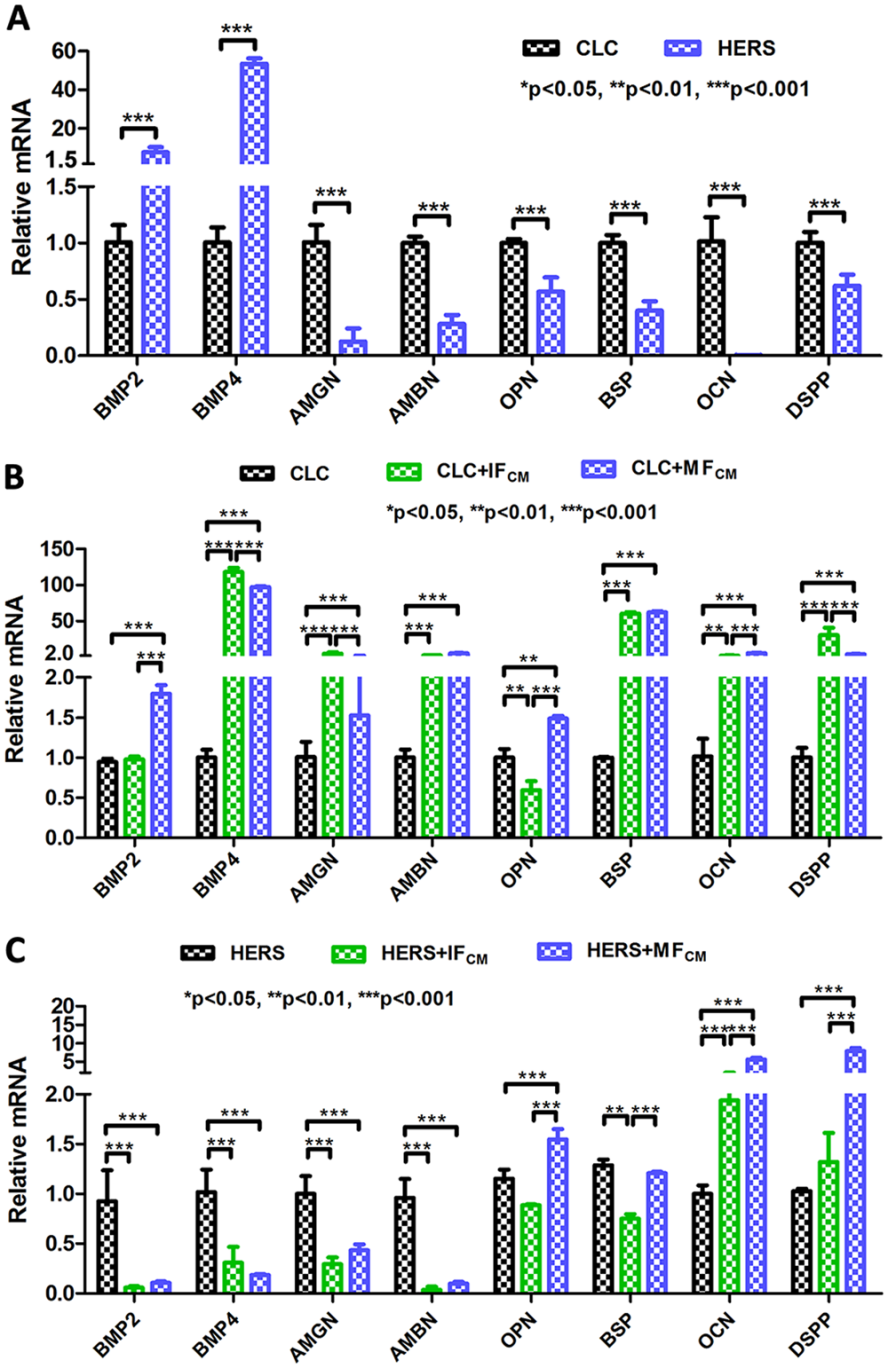
Differential expression patterns of enamel-forming and mineralization related genes between non-induced and induced CLC and HERS cells. (**A**) Non-induced CLC and HERS cells showed different gene expressions. CLC showed much lower expression of BMP2 and BMP4 but higher expression of AMGN, AMBN as well as OPN, BSP, OCN and DSPP than HERS cells. (**B**) Compared with non-induced CLC cells, IF_CM_ or MF_CM_ induced CLC cells exhibited higher expression of the detected genes, with MF_CM_ showing stronger inductive effect. (**C**) Compared with non-induced HERS cells, IF_CM_ or MF_CM_ induced HERS cells showed significantly lower expression of BMP2, BMP4, AMGN and AMBN, but higher expression of mineralization related genes, such as OPN, OCN and DSPP.

There was a significantly different gene expression profile between CLC and HERS cells. Compared with CLC, HERS cells showed higher expression of BMP2 and BMP4, but lower expression of AMGN and AMBN, which were enamel-forming relevant genes. Expressions of OPN, BSP, OCN and DSPP, which were mineralization related, were also found lower in HERS cells (Fig. 4A). After induction with IF_CM_ and MF_CM_, the expression of all investigated genes increased in CLC cells. MF_CM_ showed higher inductive effect than IF_CM_ on expression of BMP2, AMBN, OPN, BSP and OCN in CLC cells (Fig. 4B). However, BMP2, BMP4, AMBN and AMGN decreased in HERS cells induced by either IF_CM_ or MF_CM_, while expression of OPN, BSP, OCN and DSPP increased. HERS cells induced by MF_CM_ showed higher expression of mineralization relevant genes than those induced by IF_CM_ (Fig. 4C). What’s more, it was noticeable that BMP2 and BMP4 as well as AMBN and AMGN increased in IF_CM_ and MF_CM_-induced CLC cells but significantly decreased in induced HERS cells (Fig. 4B,C). Overall, CLC cells mainly expressed higher enamel-forming and mineralization related genes, which were promoted by IF_CM_ or MF_CM_, while HERS cells showed opposite expression patterns of BMP2, BMP4, AMBN and AMGN in comparison with CLC cells.

### CLC cells maintained the epithelial shape while HERS cells underwent morphology transformation when cultured with CM on inactivated tooth dentin matrix

**Figure 5:**
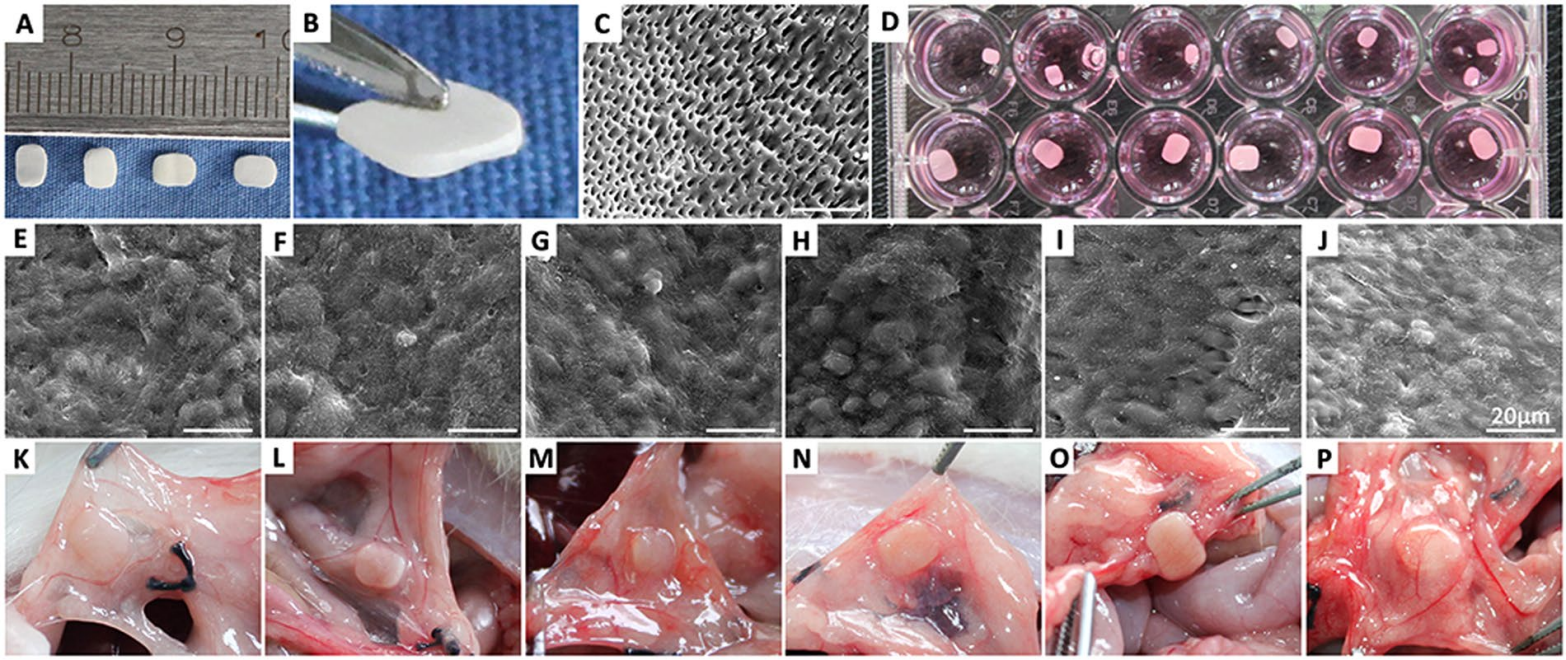
Fabrication of inactivated treated dentin matrix (iTDM), inductive culture of CLC and HERS cells on iTDM and transplantation in rat greater omentum. (**A**,**B**) iTDM were made from the root dentin of premolars extracted in clinic. (**C**) SEM examination showed complete removal of the cementum and good exposure of the dentin tubes. (**D**) CLC and HERS cells were seeded on iTDM and cultured with or without conditioned medium (CM) *in vitro* for 7 days. (**E**-**J**) SEM examination showed the morphology of CLC and HERS cells growing on iTDM. Non-induced CLC cells (**E**) and IF_CM_-induced (**F**) or MF_CM_-induced (**G**) showed similar morphology of a spheroidal shape; non-induced HERS cells (**H**) maintained the spheroidal shape while some of HERS cells lost the original characteristics and transformed into spindle-shaped cells after induction with IF_CM_ (**I**) or MF_CM_ (**J**). Fiber-like structures can be seen more prominent in MF_CM_-induced HERS cells (**J**) than IF_CM_-induced (**I**). (**K**-**P**) showed the specimen of iTMD seeded with CLC and HERS cells harvested 6 weeks after implantation in greater omentum (**K**: non-induced CLC; **L**: IF_CM_–induced CLC; **M**: MF_CM_-induced CLC; **N**: non-induced HERS; **O**: IF_CM_–induced HERS; **P**: MF_CM_-induced HERS). Scale bars: 20 μm.

Before *in vivo* transplantation, inactivated tooth dentin matrix (iTDM) was fabricated and examined by SEM (Fig. 5A–C). SEM showed the cementum was completely removed and the dentin tubes were well exposed. The porous iTDM provided as an excellent scaffold for *in vivo* transplantation of the target cells. CLC and HERS cells were seeded on the top surface of iTDMs and cultured *in vitro* for 7 days (Fig. 5D). SEM examination showed CLC and HERS cells grew well in multilayers on surface of iTDM after non-induced and induced culture *in vitro* for 7 days (Fig. 5D). CLC cells maintained the original spheroidal shape after induction by IF_CM_ or MF_CM_ (Fig. 5E–G), while some of HERS cells lost the original characteristics and transformed into spindle-shaped cells after induction with IF_CM_ or MF_CM_ (Fig. 5H–J). Fiber-like structures can be seen more prominent in MF_CM_-induced HERS cells than IF_CM_-induced.

### CLC cells give rise to enamel-like tissues while HERS cells form cementum-periodontal ligament-like structures

**Figure 6:**
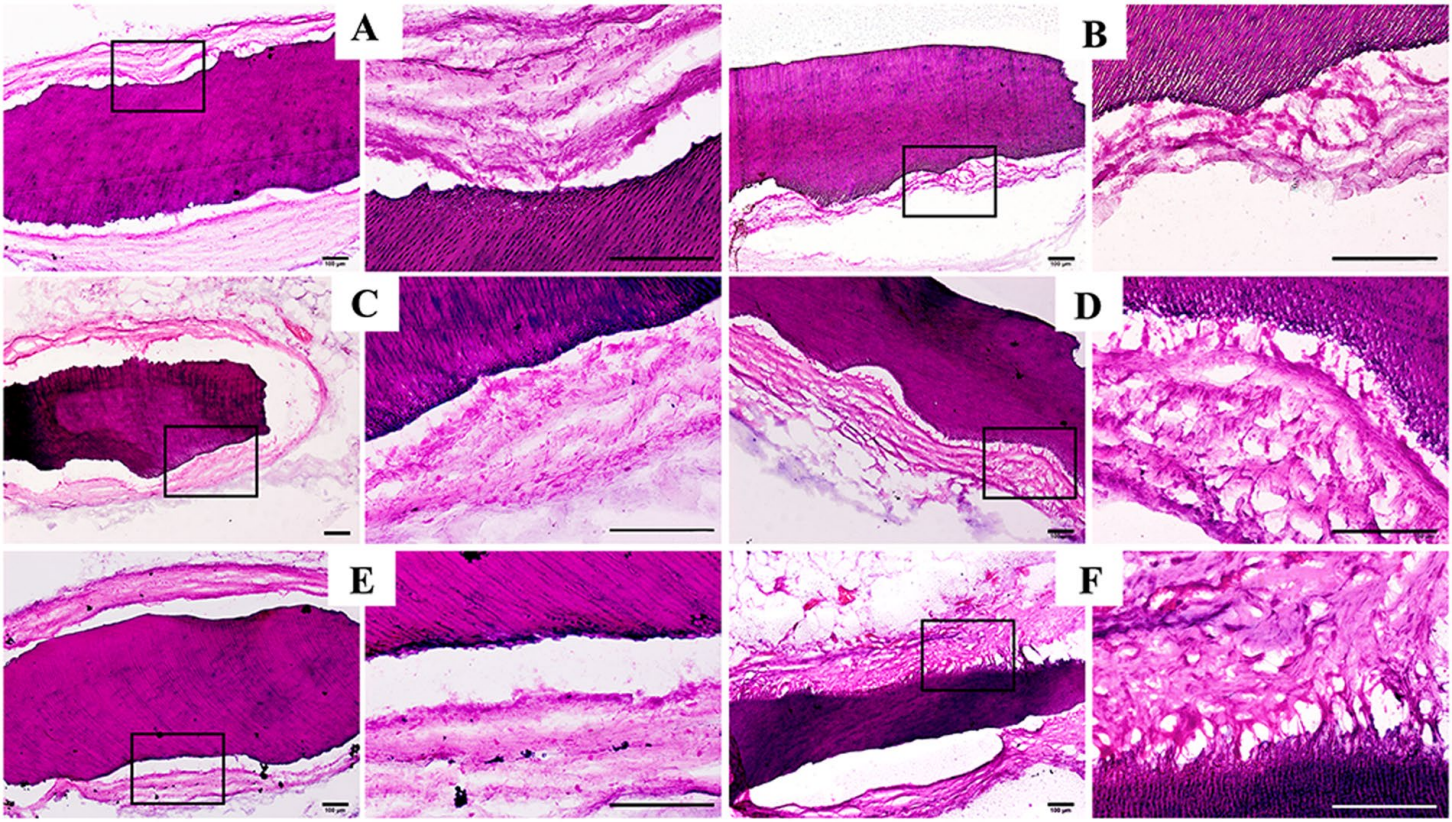
HE staining of iTDM specimen harvested from the greater omentum after demineralization, embedding and section. In CLC groups (**A**: non-induced CLC; **C**: IF_CM_–induced CLC; **E**: MF_CM_-induced CLC) no evident attachment to the surface of iTDMs was formed, while periodontal ligament-like fibers were found to attach to the surface of iTDM with an angle in HERS groups. (**B**) Non-induced HERS cells formed the least amount of fibrous attachment to iTDM, IF_CM_-induced HERS cells (**D**) formed more, and MF_CM_-induced group (**F**) formed the most. The right column was the magnification of the black box in the left column, respectively. Scale bars: 100 μm.

**Figure 7:**
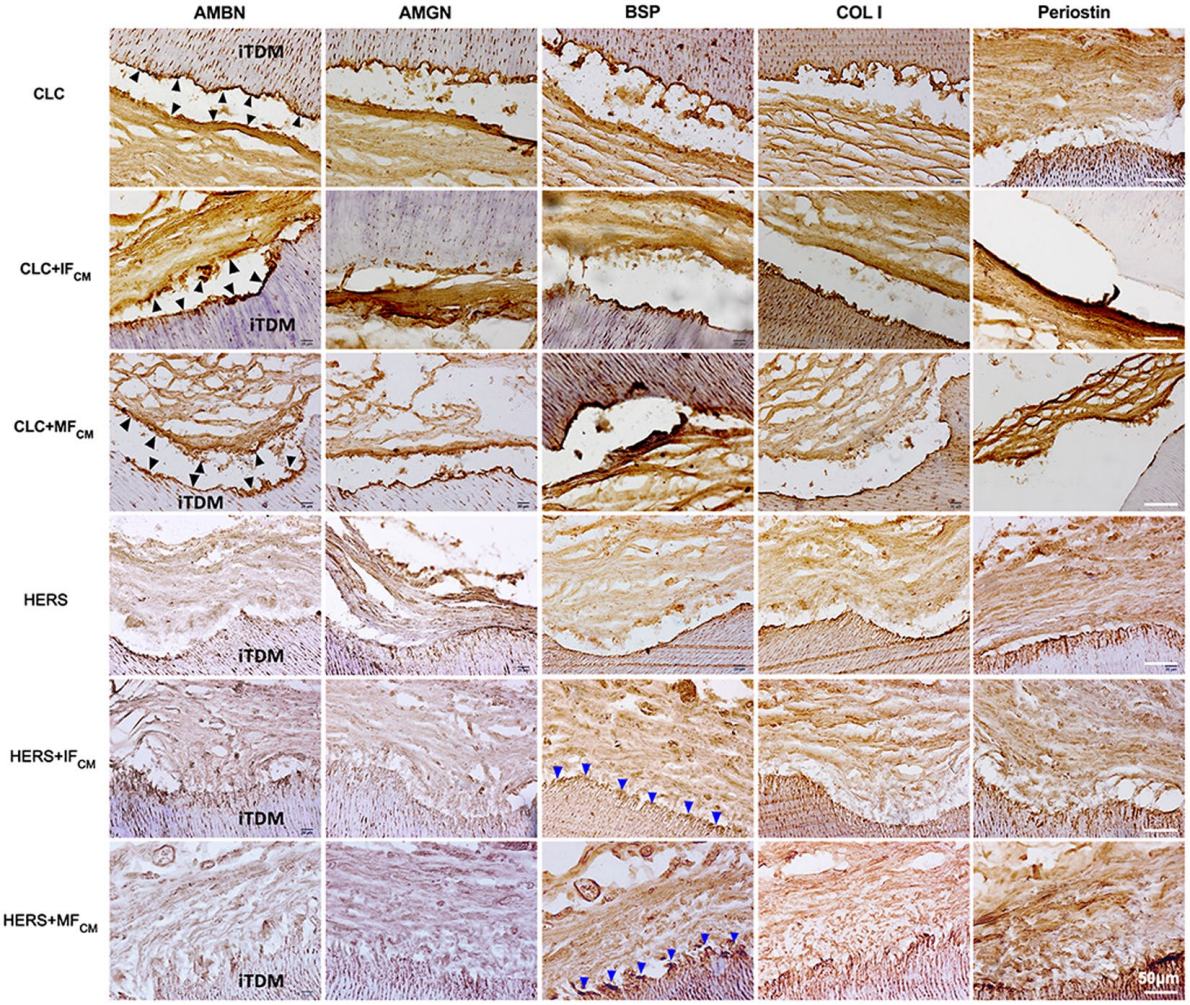
Immunohistochemistry staining of AMBN, AMGN, BSP and COL I and Periostin. AMBN, AMGN, BSP and COL I were positively stained at the interfacial layers of iTDM and the tissues opposite to iTDM in CLC groups (indicated by black arrows). These indicated that enamel-like minerals may deposit on surfaces of iTDM seeded with CLC cells. Conversely, HERS cell groups showed negative expression of AMBN and AMGN but positive for BSP, COL I and Periostin. A thin layer at the surface of iTDM in HERS cell groups (indicated by blue arrows) was positively stained for BSP, COL I and Periostin. The fibers attached to the surface layer of the iTDM were also positive for COL I and Periostin. These indicated that cementum and periodontal ligament-like tissues were formed especially in IF_CM_ and MF_CM_-induced HERS cells. Scale bars: 50 μm.

Samples were harvested after implantation in greater omentum for 6 weeks. iTDMs were encapsulated well in omentum and nourished by surrounding blood vessels (Fig. 5K–P). After demineralization, embedding and section, HE staining showed the surrounding tissues formed no evident attachment to the surface of iTDMs in CLC groups (Fig. 6A,C,E), while fiber tissues were found to attach to the surface of iTDM with a certain angle in HERS groups. HERS cells without induction formed the least fiber attachment to iTDM (Fig. 6B), while IF_CM_-induced HERS cells formed more and MF_CM_-induced group formed the most. The arrangement of the attached fibers resembled to the periodontal ligament fibers (Fig. 6D,F). Further immunohistochemistry staining showed AMBN, AMGN, BSP and COL I were positively stained at the interfacial layers of iTDM and the fiber tissues opposite to iTDM in CLC groups (Fig. 7 indicated by black arrows). AMBN and AMGN were abundant and critical in enamel. The positive staining of AMBN and AMGN indicated enamel-like minerals were deposited on surfaces of iTDMs seeded with CLC cells. On the contrary, HERS groups showed negative expression of AMBN and AMGN but positive for BSP, COL I and Periostin. As indicated by blue arrows in Fig. 7, a thin layer at the surface of iTDM, to which the fibers attached, was positively stained for BSP, COL I and Periostin. The attaching fibers were also positive for COL I and Periostin. These suggested that cementum-periodontal ligament like tissues were formed in HERS groups, especially in IF_CM_ and MF_CM_ induced ones.

## Discussion

In this study, we isolated and induced CLC and HERS cells using conditioned media collected from the mesenchymal IF and MF cells. Both *in vitro* and *in vivo* studies demonstrated that CLC and HERS cells showed distinct differentiation patterns under the inductions from dental follicle cells.

Assessments in morphology, proliferation ability, mineral formation and gene expression *in vitro* revealed distinct differentiation between CLC and HERS cells. Our results showed that CLC maintained the epithelial shape after induction while a part of HERS cells underwent morphological transformation(Fig. 2A–F). Differences in cell proliferation (Fig. 2G–I), gene expression (Fig. 4) and the mineralization ability (Fig. 3) were also found between CLC and HERS cells. Given that the biological performances mentioned above are closely related to the cell differentiation, it can be demonstrated that CLC and HERS cells differentiated in different ways. Gene expression analysis found that CLC cells showed higher expression of AMBN and AMGN (Fig. 4A), which are critical for enamel formation and deletion of them would result in abnormity or absence of the enamel^13,14^. It was also found that the mineralization related genes, such as BSP, OCN and OPN, were significantly higher in CLC cells than the HERS cells (Fig. 4A). These suggested that CLC cells were able to differentiate into ameloblasts, which give rise to the enamel matrix, and the increased mineralization ability may contribute to the maturation of the enamel. IF_CM_ and MF_CM_ had significant inductive effect to promote the differentiation of CLC into ameloblasts (Fig. 4B). In contrast, non-induced HERS cells showed higher expression of BMP2 and BMP4 than CLC cells (Fig. 4A), and induced HERS cells expressed lower BMP2 and BMP4 than non-induced HERS cells (Fig. 4C). These suggested that BMP2 and BMP4 might be critical signals reregulating the distinct differentiation between CLC and HERS cells in response to induction from dental follicle cells. Yamamoto *et al*. also found that HERS cells express BMP2 and BMP4, which could regulate the development of the root^11,15–17^. In addition, Somerman and Bosshardt *et al*. claimed that HERS cells could produce BSP, OPN and COLI, which are relevant to the mineralization of cementum^16,17^. This is consistent with the results of the present study, which found that induced HERS cell expressed higher mineralization related genes, such as BSP, OPN and OCN. These indicated that HERS cells possibly were able to transform into cementoblasts, which produce the cementum. The distinct forms of the minerals revealed by the alizarin red staining (Fig. 3) could also be explained by the different differentiation ways between CLC and HERS cells. The massive minerals formed in CLC cells but granular ones in HERS cells might correspond to the different structures and components between enamel and cementum tissues, respectively.

CLC cells could differentiate into ameleoblasts and give rise to enamel-like tissues. As shown by Fig. 7 (indicated by black arrows), the positive staining of AMBN, AMGN and COL I indicated that enamel-like minerals were deposited on the interfacial layers of iTDM and the tissues opposite to iTDM in all CLC groups. Previous studies have found that there is an epithelial stem cell niche in the labial cervical loop of rodent incisors. The self-renewal and differentiation of the epithelial stem cells are responsible for the continuous generation of the enamel, and the dental mesenchyme plays crucial roles in influencing the stem cell niche^1,18^. The proliferation and differentiation of the stem cells in cervical loops is controlled by an integrated regulatory network consisting of Activin, bone morphogenetic protein (BMP), and fibroblast growth factor (FGF)^19,20^. Mesenchymal FGF3 from dental papillae stimulates epithelial stem cell proliferation, but BMP4 represses FGF3 expression and promotes ameloblast differentiation. In turn, Activin, which is strongly expressed in labial dental follicles, inhibits the repressive effect of BMP4 and restricts FGF3 expression to apical end of mesenchyme in dental papillae, resulting in increased stem cell proliferation and a large, labial cervical loop structure. Thus, FGF3 is necessary for maintenance of the epithelial stem cell pool that provides a continuous supply of ameloblast progenitors. On the contrary, the expression of FGF3 in dental follicles is obviously less than that in dental papillae. Therefore, based on previous studies^19,20^, it can be drawn that the dental follicles promote differentiation of the progenitors into ameloblasts under the inhibitive effect of BMP4 on FGF3. These findings are consistent with our study, which found that CLC owned the ability to maintain the epithelial characteristic and to differente into enamel–forming ameloblasts, and the differentiation was promoted by IF_CM_ and MF_CM_.

HERS cells directly take part in the formation of cementum-periodontal ligament-like structures. Although the fate of HERS cells has been controversial, some scholars believe that HERS cells are able to transform into periodontal ligament fibroblasts and cementoblasts in the development of the periodontal tissues^21^. A study using K14-Cre R26R mouse model also indicated HERS cells participate in the formation of cementum and periodontal ligament fibers^22^. In agreement with previous studies, our results verified that HERS cells could undergo morphology transformation *in vitro* and form cementum-periodontal ligament-like structures *in vivo*. Many researchers believed that this process is achieved by the epithelial-mesenchymal transformation (EMT)^18,23^, which is the transformation of epithelial cells into mesenchymal cells^24,25^. Most of the EMT processes are regulated by transforming growth factor-β (TGF-β) and basic fibroblast growth factor (bFGF)^26^. Recent studies also showed that TGF-β 1 and bFGF2 could regulate the EMT of HERS cells via MAPK/ERK signaling pathway^27,28^. bFGF 2 could induce HERS cells to transform towards cementoblasts while TGF induce HERS cells transform towards periodontal ligamental fibroblasts. bFGF 2 is widely expressed at the dental basement membrane as well as in dental follicle and dental papilla, and it could promote the differentiation, proliferation and migration of cementoblasts^29^. Meanwhile, TGF-β 1 could induce the differentiation of periodontal ligament cells and maintain the physiological function of the periodontal complex^30^. After completion of the root development, the residual HERS cells in the periodontal ligament become the epithelial rests of Malassez (ERM), which take part in maintaining the homeostasis of the periodontium and the restoration or regeneration after destruction^12,31^. Therefore, under the stimulation of cellular factors from the mesenchyme, HERS cells are able to differentiate into cementoblasts and periodontal ligament cells participating in the formation of cementum and periodontal ligament.

Nevertheless, it is still unclear what cellular and molecular mechanisms are involved in the different differentiation patterns between CLC and HERS cells and how dental follicle cells correlate. Further investigations focusing on the cell-autonomous and non-cell-autonomous mechanisms of CLC and HERS cells would be beneficial for unveiling the myth of different development modes between rodent incisors and molars. Studies of dental follicle cells on how they regulate the biological performances of the two epithelial cells, such as the differential expressions of BMP2 and BMP4, are also necessary.

## Conclusion

CLC and HERS cells present distinct differentiation patterns under the inductions from the dental follicle cells. CLC cells could maintain the epithelial characteristics and differentiate to form enamel-like tissues, while HERS cells could transform and directly participate in the formation of cementum and periodontal ligmament-like structures. Nevertheless, further studies are needed to unveil the underlying cellular and molecular mechanisms.

## Materials and Methods

All the experimental protocols and animal experimental procedures employed in this study were approved by the Ethics Committee of West China School of Stomatology, Sichuan University, China, and all methods were performed in accordance with the relevant guidelines and regulations.

### Cell isolation, purification and identification

**Figure 8:**
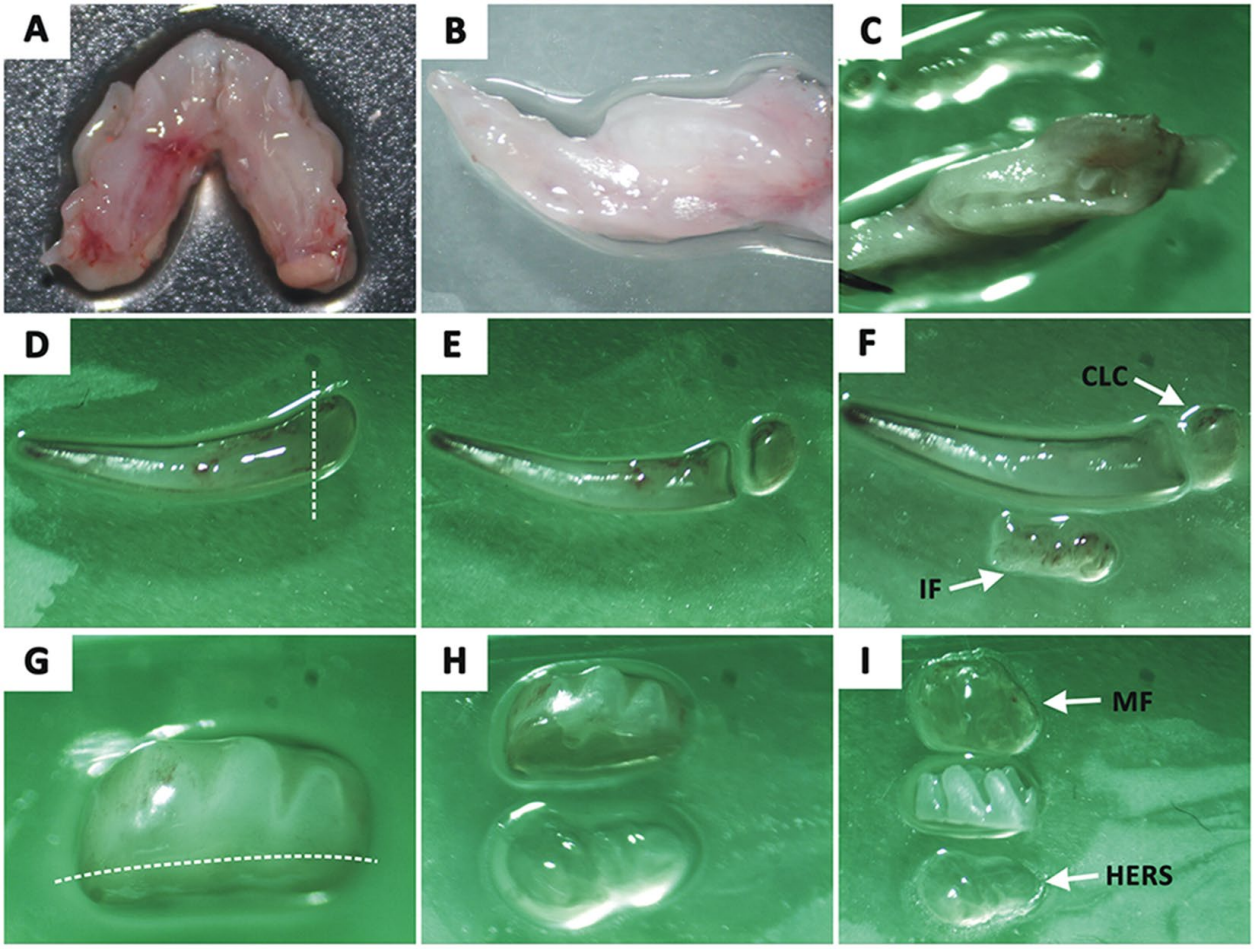
Cell isolation. Mandibles were dissected from post-natal 7-day Sprauge-Dawley rats (**A**,**B**) and the gingival epithelium covering the tooth germs were removed (**C**). Incisors and the first molars were isolated (**D**,**G**). Then, cervical loop cells (CLC) and incisor dental follicle cells (IF) were isolated from mandiblular incisor (**E**,**F**), while HERS cells (HERS) and molar dental follicle cells (MF) were obtained from molar germs (**H**, **I**) respectively.

Cervical loop cells (CLC), incisor dental follicle cells (IF), HERS cells (HERS), molar dental follicle cells (MF) were isolated from incisor and molar germs of post-natal (PN) 7-day Sprauge-Dawley (SD) rats referring to a modified method as previously described^11,32^. Briefly, postnatal 7-day old SD rats were euthanized by cervical dislocation under over-dose anesthesia. The mandibles were dissected (Fig. 8A–C) and then the incisor and first molar germs were isolated with the aid of a stereomicroscope (Fig. 8D,G). To obtain CLC, the labial apical end tissue of the incisor germs was dissected (Fig. 8E). Then, the thin layers of the dental follicle above the incisor germs were gently separated to obtain IF (Fig. 8F). For HERS cell and MF cell isolation, the apical end tissues of molar germs and the dental follicle around the molar germs were dissected and collected referring to the procedures of cervical loop and IF isolation (Fig. 8G–I). The cervical loop, IF, HERS or MF tissues were then minced into pieces, and digested with a mixture of 625 U/mL type I collagenase (Sigma–Aldrich, USA) and 2.4 U/mL Dispase II (Sigma–Aldrich, USA) at 37 °C, 1 h for CLC and HERS tissues while 0.5 h for IF and MF tissues. After centrifugation at 1200 rpm for 5 minutes, the CLC and HERS cells were re-suspended and cultured in epithelial cell medium (EpiCM; ScienCell, USA) consisting of basal medium, 2% fetal bovine serum (FBS; ScienCell, USA), 1% epithelial cell growth supplement (EpiCGS; ScienCell, USA), and 1% penicillin/streptomycin solution (P/S; ScienCell, USA). IF and MF cells were re-suspended and cultured with α-MEM supplemented with 15% FBS (Hyclone, USA), 1% P/S (Solarbio, China). Cells were incubated at 37 °C in a humidified atmosphere with 5% CO_2_. The medium was changed every 2 days. After the cells grow and fuse by 70%, a method of differential digestion was performed to purify the targeting cells using trypsin/EDTA (Millipore, USA) according the procedures described previously^27^. Purified cells were further identified by immunofluorescence staining. CLC and HERS cells were assessed for positive immunofluorescent staining with mouse anti-CK14 antibody (1:800; Abcam, UK) and negative staining with mouse anti-vimentin antibody, while IF and MF cells were assessed for negative anti-CK-14 staining and positive anti-vimentin staining. Cells that met these criteria were subsequently used in the experiments.

### Inducing culture of CLC and HERS cells with IF or MF cell derived conditioned medium

Conditioned media (CM) were collected from culture of IF and MF cells respectively, using the methods described as following. When the 3^rd^ passages of IF and MF cells grow and fuse by 70%, the medium was collected and new medium was added to the cells every 2 days. The collected medium from IF and MF cell culture was filtrated with films and then mixed with EPiCM in the proportion of 1:1 to make the CM: the IF cell derived CM (IF_CM_) and MF cell derived CM (MF_CM)_. The purified CLC and HERS cells were then cultured with CM as shown in Table 1.

**Table 1:** Inductive culture of CLC and HERS cells with conditioned midiumtim.

Group	Cells	Culture medium
1	CLC	EpiCM
2	CLC	EpiCM + IF_CM_ (1:1)
3	CLC	EpiCM + MF_CM_ (1:1)
4	HERS	EpiCM
5	HERS	EpiCM + IF_CM_ (1:1)
6	HERS	EpiCM + MF_CM_ (1:1)

### Morphological observation

To investigate the effect of IF_CM_ and MF_CM_ on the morphological changes of CLC and HERS cells during the inducing culture, we observed and photographed the morphological difference between induced and non-induced CLC and HERS cells under a phase-contrast inverted microscope (Leica DMI 6000, Germany) after 7 days of culture. The experiment was repeated at least three times.

### Cell proliferation analysis

A Cell Counting Kit-8 (CCK-8, Dojindo, Japan) was used to quantitatively evaluate cell proliferation. CLC and HERS cells were seeded in a 96-well plate with an initial density of 5 × 10^4^/well and cultured with medium as shown in Table 1. After cells were cultured for 1d, 2d, 3d, 4d, 5d, 6d or 7d, the culture medium of each well was replaced by 120 μl original medium, which contained 12 μl CCK-8, and the plates were incubating at 37 °C for 4 h. Then 100 μl of the above solution was taken from each well and added to one well of a new 96-well plate. At least three parallel replicates were prepared. The optical density (OD) value at 450 nm was determined using a spectropho-tometer (Thermo VARIOSKAN FLASH, Thermo, USA).

### Mineralization ability assay

Alizarin red staining was used to assess the mineral formation of CLC and HERS cells which were cultured under the conditions listed in Table 1 on a 6-well plate. After 7 days culture, cells were washed twice in PBS and fixed in 4% paraformaldehyde for 10 minutes and then incubated in 0.1% alizarin red solution (Sigma, USA) in Trise HCl (pH 8.3) at 37 °C for 30 minutes. After being washed twice in PBS, cells were photographed under a light microscope (Leica DMI 6000, Germany).

### Quantitative real-time PCR analysis

Quantitative real-time PCR (qRT-PCR) analysis was used to detect the related gene expression in CLC and HERS cells that were cultured under the conditions listed in Table 1. After 7 days of culture, cells were harvested and total RNA extraction was performed using RNAiso plus (TaKaRa, Dalian). cDNA synthesis was performed with SYBR® Premix Ex Taq II (Perfect Real Time kit; TaKaRa, Dalian). Experiments were performed in triplicates according to the manufacturer’s instructions. Sequences of the gene-specific primers synthesized by TaKaRa are listed in Table 2. Normalized gene expression values for each sample were calculated as the ratio of expression of mRNA for the target genes to the expression of mRNA for β-actin.

**Table 2:** Primer sequences of target genes.

Gene	Primers (5′–3′)	Fragment(bp)	GeneBank No.
BMP2	F gacatccactccacaaacgaga	189	NM_017178.1
	R gtcattccaccccacatcact		
BMP4	F ctatttcgggagcaggtggac	199	NM_012827.2
	R ccagtagtcgtgtgatgaggtgt		
AMGN	F agcttttgctatgcccctacc	217	U51195.1
	R gatgaggctgaagggtgtgact		
AMBN	F ctgctcctgttcctgtcccta	246	NM_012900.1
	R gcttcccaactgtctcattgtc		
OPN	F gaggctatcaaggtcatcccagt	290	M14656.1
	R ctggccctctgcttatactcctt		
BSP	F gaaagagcagcacggttgagta	167	DQ213013.1
	R cgtcctcataagctcggtaagtg		
OCN	F cctctctctgctcactctgctg	166	NM_199173.3
	R accttactgccctcctgcttg		
DSPP	F atgggacacagcaggataggc	244	NM_012790.2
	R cacttccgtcacttccgttagac		
β-ACTIN	F acggtcaggtcatcactatcg	155	NM_031144.3
	R ggcatagaggtctttacggatg		

### Fabrication of inactivated treated dentin matrix (iTDM)

For *in vivo* evaluation of the differentiation of induced and non-induced CLC and HERS cells, iTDM was used as the scaffold for *in vivo* transplantation. iTDM was fabricated referring to the method modified according to the procedures described previously^33–35^. Briefly, ten premolars were harvested from five healthy patients who required tooth removal for orthodontic treatment. Periodontal ligament tissues were completely scraped away, and the outer cementum, inner dental pulp tissues, predentin and partial root dentins were removed by grinding. The resulting dentin matrix was cut into pieces and grinded into regular shape as shown in Fig. 7A,B. Gradient de-mineralization of the dentin matrix was further performed by treating with EDTA solution of gradient concentrations according to detailed procedures in previous studies^33–35^. Afterwards, the treated dentin matrix was inactivated by moist heat sterilization and dried for further experiments. Morphological observation of the iTDM was observed by scanning electron microscope (SEM) (Inspect F, FEI, Netherlands).

### *In vivo* transplantation and histological analysis

To analyze the CLC and HERS cell differentiation *in vivo* after induction with conditioned medium, cells were seeded on the top surfaces of iTDM and then transplanted in SD rat great omentum. Briefly, iTDMs were first placed in 24-well plate, then seeded with an initial amount of 5 × 10^5^ CLC and HERS cells respectively, and cultured *in vitro* for 7 days according to Table 1 with changing of medium every 2 days. Afterwards, SEM examination was performed to detect the growth and morphology of cells on iTDMs, and implantation of iTDMs with cells in rat great omentum was carried out under deep anesthesia. iTDM without cell seeding was used as blank control. For each sample, at least three replicates were made. 4 weeks later, implants were harvested from the omentum under deep anesthesia. Samples were then fixed with 4% paraformaldehyde overnight at 4 °C, demineralized with 10% EDTA (pH 8.0) and embedded in paraffin. Sections were made and stained with hematoxylin and eosin (H&E). Immunohistochemical staining was also performed using antibodies for AMBN (Millipore, USA), AMGN (Millipore, USA), BSP (Abcam, UK), COL I (Abcam, UK) and periostin (Abcam, UK) at a dilution of 1:200 according to the manufacturers’ protocol. Histological images were taken under microscope (Olympus, Japan).

### Statistical analysis

Data are presented as the mean ± SD from at least three independent experiments. Statistical comparison was performed using Student *t* test. SPSS 17 software (SPSS, USA) was used to perform statistical analysis and a *p* ≤ 0.05 was consider statistically significant.
